# Global Epidemiology and Antimicrobial Resistance of Klebsiella Pneumoniae Carbapenemase (KPC)-Producing Gram-Negative Clinical Isolates: A Review

**DOI:** 10.3390/microorganisms13071697

**Published:** 2025-07-19

**Authors:** Matthew E. Falagas, Christina-Maria Asimotou, Maria Zidrou, Dimitrios S. Kontogiannis, Charalampos Filippou

**Affiliations:** 1 Alfa Institute of Biomedical Sciences, 9 Neapoleos Street, Marousi, 151 23 Athens, Greeced.kontogiannis@aibs.gr (D.S.K.); 2 School of Medicine, European University Cyprus, 6 Diogenous Str., Egkomi, Nicosia 2404, Cyprus; c.filippou@euc.ac.cy; 3 Department of Medicine, Tufts University School of Medicine, Boston, 145 Harrison Ave, Boston, MA 02111, USA

**Keywords:** *Klebsiella pneumoniae* carbapenemase (KPC), Gram-negative, global epidemiology, global dissemination, β-lactamase

## Abstract

*Klebsiella pneumoniae* carbapenemases (KPCs) are a group of class A β-lactamases of Gram-negative bacteria leading to difficult-to-treat infections. We evaluated the global epidemiology of KPC-producing Gram-negative clinical isolates. A systematic search of six databases (Cochrane Library, Embase, Google Scholar, PubMed, Scopus, and Web of Science) was conducted. Extracted data were tabulated and evaluated. After screening 1993 articles, 119 were included in the study. The included studies originated from Asia (*n* = 49), Europe (*n* = 29), North America (*n* = 14), South America (*n* = 11), and Africa (*n* = 3); 13 studies were multicontinental. The most commonly reported KPC-producing species were *Klebsiella pneumoniae* (96 studies) and *Escherichia coli* (52 studies), followed by *Enterobacter cloacae* (31), *Citrobacter* spp. (24), *Klebsiella oxytoca* (23), *Serratia* spp. (15), *Enterobacter* spp. (15), *Acinetobacter baumannii* complex (13), *Providencia* spp. (11), *Morganella* spp. (11), *Klebsiella aerogenes* (9), *Pseudomonas aeruginosa* (8), *Raoultella* spp. (8), *Proteus* spp. (8), and *Enterobacter aerogenes* (6). Among the studies with specific *bla*_KPC_ gene detection, 52/57 (91%) reported the isolation of *bla*_KPC-2_ and 26/57 (46%) reported *bla*_KPC-3_. The antimicrobial resistance of the studied KPC-producing isolates was the lowest for ceftazidime–avibactam (0–4%). Resistance to polymyxins, tigecycline, and trimethoprim–sulfamethoxazole in the evaluated studies was 4–80%, 0–73%, and 5.6–100%, respectively. **Conclusions:** The findings presented in this work indicate that KPC-producing Gram-negative bacteria have spread globally across all continents. Implementing proper infection control measures, antimicrobial stewardship programs, and enhanced surveillance is crucial.

## 1. Introduction

Antimicrobial resistance increases the mortality of patients with various types of infections [1]. If no appropriate measures are taken to combat this problem, deaths due to infections with antimicrobial resistance will continue to rise globally in the coming years [2,3]. In addition, antimicrobial resistance considerably increases the length of hospital stay and healthcare-associated costs for all countries [2]. 

One of the basic microbial mechanisms contributing to the development of antimicrobial resistance is the production of β-lactamases [4]. Beta-lactamases hydrolyze the β-lactam ring of antibiotics. They are grouped as class A, B, C, and D based on the Ambler classification [4,5]. Classes A, C, and D use serine at their active site, whereas class B enzymes (metallo-β-lactamases) require zinc. A subset of class A β-lactamases, specifically *Klebsiella pneumoniae* carbapenemase (KPC), is significant due to its global dissemination and the increased incidence of opportunistic infections in immunocompromised patients caused by bacteria, mainly *Klebsiella pneumoniae* (*K. pneumoniae*) that harbor KPC [4,6]. The worldwide spread of KPC has been linked to the dissemination of a main clone of *K. pneumoniae* [sequence type (ST) 258] and a single-locus variant of ST258, specifically ST512 that is prevalent in Italy, Colombia, and Israel [7,8,9]. In Asia, most specifically in China, another variant of ST258, specifically ST11, is mostly reported among *bla*_KPC_-harboring *K. pneumoniae* isolates [8,10]. It is ultimately known that ST258 and its variants are the principal clones accounting for the majority of KPC-producing *K. pneumoniae* globally [9]. ST307 is another globally spread clone, which raises concerns among the scientific community [11]. This clone has been found in Greece, Italy, and Spain. In addition, the clones ST340 and ST437 have caused frequent clinical outbreaks in Brazil and Greece [9]. Other successfully KPC-producing pathogens nowadays include *Enterobacterales*, such as *Escherichia coli*, with the clone ST131 being the most dominant worldwide along with the ST258 *K. pneumoniae* [12]. Another *E. coli* clone, ST410, has been rising in China [12]. Other *Enterobacterales* that produce KPCs are *Klebsiella oxytoca*, *Enterobacter* spp., and *Serratia* spp., as well as lactose-non-fermenting Gram-negative bacilli, including *Pseudomonas* spp., and *Acinetobacter baumannii* [13].

Infections caused by KPC-producing pathogens are associated with considerable morbidity and mortality [14]. Two factors contribute to this result. First, a significant proportion of infections due to KPC-producing pathogens occur in patients in healthcare settings who already have considerable comorbidities. Second, the therapeutic options for patients with such infections are limited [15]. Subsequently, the outcome is often unfavorable, particularly for patients with severe infections and significant comorbidities.

Previous studies have evaluated the distribution of carbapenemase-producing isolates [16,17,18,19]; however, limited data exist regarding the global epidemiology of KPC-producing Gram-negative pathogens, particularly regarding recent developments. The scope of this review is to address the information gap by gathering and evaluating all relevant and recent bibliographical data on this topic.

## 2. Methods

### 2.1. Objectives

This review evaluated the global epidemiology of KPC-producing Gram-negative bacteria and their resistance to various antimicrobial agents.

### 2.2. Eligibility Criteria

Studies that included Gram-negative clinical pathogens in their analyses were eligible for inclusion in this study. There were no limitations regarding the language, date, geographical location, publication journal, age, gender, and patient settings (hospitalized or not). Reports from the gray literature, such as conference abstracts, were excluded from further analysis in the screening process. Studies that included fewer than five clinical isolates were excluded.

Only studies confirming the presence of KPC genes with the polymerase chain reaction (PCR) method and antimicrobial resistance based on the Clinical and Laboratory Standards Institute (CLSI) and European Committee on Antimicrobial Susceptibility Testing (EUCAST) recommendations were eligible for inclusion.

### 2.3. Search Strategy

On 20 February 2025, specific search strings using combinations of the terms “*Klebsiella pneumoniae* carbapenemase”, “KPC-producing”, “carbapenemase”, “prevalence”, Gram-negative”, “worldwide”, and “global” were applied in six resources (Cochrane Library, Web of Science, Embase, PubMed, Google Scholar, and Scopus) for the identification of relevant articles. Supplementary Table S1 presents the detailed search strings used.

### 2.4. Selection Process

Identified studies from the six resources were deduplicated using the SR Accelerator software. Two reviewers (among CMA, MZ, and DSK) screened these studies, first by title and abstract, and then by full text. Discrepancies between the reviewers’ findings were resolved in meetings with a senior author (MEF). All the retrieved articles deemed relevant were included in the analysis. Additionally, one reviewer (CMA, MZ, or DSK) examined the references of pertinent review articles related to the topic to identify any additional reports that might have been missed.

### 2.5. Data Extraction

Two reviewers (among CMA, MZ, and DSK) tabulated the following data in a spreadsheet: first author, year, continent, country, period of isolation, population characteristics (age, hospital ward), samples used for bacterial detection, species of isolates, and gene types of KPC. The proportion of KPC-producing pathogens was evaluated according to the available data of each article and expressed in fractions and percentages. Discrepancies were resolved by consensus with a senior author (MEF). When reported, the proportion of KPC-producing bacteria resistant to various antimicrobial agents was recorded (as a percentage).

## 3. Results

### 3.1. Selection of Relevant Articles

**Figure 1:**
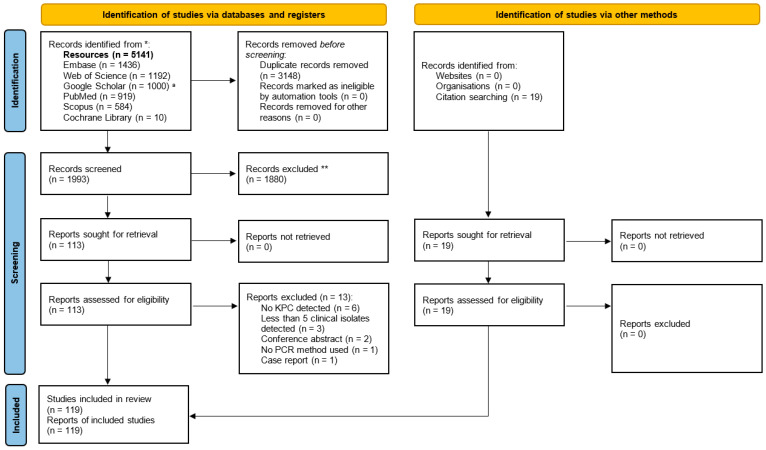
^a^ There were 5120 results in total but only the first 1000 results could be accessed with Google Scholar. * Consider, if feasible, reporting the number of records identified from each database or register searched (rather than the total number across all databases/registers). ** If automation tools were used, indicate how many records were excluded by a human and how many were excluded by automation tools.

Figure 1 presents the identification, screening, and inclusion of articles in the PRISMA (Preferred Reporting Items for Systematic Reviews and Meta-Analyses) flow diagram. After removing 3148 duplicates, 1993 articles remained for screening, and, finally, 119 articles were eligible for inclusion in this review.

### 3.2. Results of Individual Studies

In Table 1, a total of 119 studies were included. Of these, 103 were classified as observational or clinical outbreak studies, including 32 that were limited in scope (small sample size, studies conducted at a single institution, or restricted timeframe). Additionally, 12 studies were categorized as multi-country surveillance and 4 were categorized as systematic surveillance studies. These classifications highlight the methodological diversity in the reporting and monitoring of *Klebsiella pneumoniae carbapenemase* (KPC) detection globally. The data on the 119 included studies (first author, year, country, continent, period of isolation), the species identified, and the proportion of the detected *bla*_KPC_ genes via PCR are presented. A total of 49 studies originated from Asia (25 from China [20,21,22,23,24,25,26,27,28,29,30,31,32,33,34,35,36,37,38,39,40,41,42,43,44], 4 from Israel [45,46,47,48], 3 from Nepal [49,50,51], 3 from Singapore [52,53,54], 3 from Taiwan [55,56,57], 2 from South Korea [58,59], and 2 from Vietnam [60,61]), while 1 study came from each of the following countries: Saudi Arabia [62], India [63], Iran [64], Iraq [64], Venezuela [65], Turkey [66], and Russia [67]. A total of 29 studies were from Europe (4 from Greece [68,69,70,71], 4 from Italy [72,73,74,75], 3 from Poland [76,77,78], and 2 from Hungary [79,80]), while 1 study from each of the following countries: Austria [81], Belgium [82], Bulgaria [83], Denmark [84], Finland [85], France [86], Ireland [87], the Netherlands [88], Norway [89], Romania [90], and Spain [91], as well as 5 including multiple European countries [11,92,93,94,95]). A total of 14 studies were from North America (12 from the USA [96,97,98,99,100,101,102,103,104,105,106,107] and 2 from Canada [108,109]). A total of 11 studies were from South America (6 from Brazil [110,111,112,113,114,115], from 2 Colombia [116,117], and 1 from each of the following countries: Argentina [118], Chile [119], and Ecuador [120]). Three studies were from Africa (one each from Egypt [121], Nigeria [122], and Uganda [123]). Additionally, four global surveillance studies [124,125,126,127] and nine studies spanning multiple continents [128,129,130,131,132,133,134,135,136] were included. 

The isolation period of strains ranged from 1997 [96] to 2024 [73,74] across the included studies. Among the 83 out of the 118 studies (70.3%) that reported sample sources, bloodstream isolates were most common (72 studies), followed by urine (58), respiratory tract (33), sputum (27), wound swabs (25), rectal swabs (17), skin or soft tissue (18), catheter-related (13), abdominal (14), bile (9), pus (8), and ascitic fluid (5). 

In terms of isolated organisms, *K. pneumoniae* was reported in 96 studies, *Escherichia coli* in 52, *Enterobacter cloacae* in 31, *Citrobacter* spp. in 24, *Klebsiella oxytoca* in 23, *Serratia* spp. in 15, *Enterobacter* spp. in 15, *Acinetobacter baumannii* complex in 13, *Providencia* spp. in 11, *Morganella* spp. in 11, *Klebsiella aerogenes* in 9, *Pseudomonas aeruginosa* in 8, *Raoultella* spp. in 8, *Proteus* spp. in 8, and *Enterobacter aerogenes* in 6 studies. A few studies also isolated other genera (e.g., *Salmonella* spp., *Cronobacter sakazakii*, *Achromobacter denitrificans*, *Klebsiella quasipneumoniae*, *Klebsiella variicola*, *Pantoea* spp., *Kluyvera* spp., *Pluralibacter gergoviae*, *Hafnia alvei*).

Fifty-eight studies included data on the detection of specific *bla*_KPC_ genes. The majority [53/58 (91%)] reported the isolation of *bla*_KPC-2_. About half [27/58 (47%)] reported the isolation of *bla*_KPC-3_. Other *bla*_KPC_ genes were also detected, such as *bla*_KPC-4_, *bla*_KPC-6_, *bla*_KPC-8_, *bla*_KPC-9_, *bla*_KPC-11_, *bla*_KPC-12_, *bla*_KPC-17_, *bla*_KPC-18_, *bla*_KPC-20_, *bla*_KPC-29_, *bla*_KPC-30_, *bla*_KPC-31_, *bla*_KPC-36_, *bla*_KPC-46_, and *bla*_KPC-66_.

In Table 2 and the Supplementary Table S2, the antimicrobial resistance of KPC-producing isolates is presented. According to the included studies that provided relevant data, resistance was lowest for polymyxins (colistin or polymyxin B) (ranging from 4 to 80% across studies) and for ceftazidime–avibactam (ranging from 0 to 4%). Some studies also reported relatively low resistance rates for tigecycline (ranging from 0 to 73%) and for trimethoprim–sulfamethoxazole in some others (ranging from 5.6 to 100%). However, antimicrobial resistance is high for other classes of antimicrobial agents, including third-generation cephalosporins (cefotaxime, ceftriaxone, and ceftazidime), cefepime (a fourth-generation cephalosporin), piperacillin–tazobactam (an antipseudomonal antimicrobial agent), carbapenems (imipenem, meropenem, and ertapenem), and fluoroquinolones (ciprofloxacin and levofloxacin), reaching up to 100% (Table 2 and Supplementary Table S2).

## 4. Discussion

The results of our study demonstrate the widespread occurrence of infections caused by KPC-producing pathogens in most parts of the world. The first published case of infection due to a KPC-producing organism was from the US in 2001, describing the molecular characterization of KPC-1, a novel group 2f, class A, carbapenem-hydrolyzing β-lactamase, from a pathogen isolated from a patient in 1996 [138,139]. Infections due to KPC-producing pathogens have now been disseminated globally. 

Infection control deficiencies and pressure for the emergence of antimicrobial resistance, combined with frequent international travel, have led to this new global public health problem [140]. Infections by KPC-producing pathogens are now common in Europe, Asia [including India and China, frequently in conjunction with the metallo-β-lactamase (MBL) antimicrobial resistance mechanism, especially the presence of the New Delhi metallo-β-lactamase (NDM)], North America, South America (especially in Brazil and Colombia), the Middle East (frequently in conjunction with the OXA-48 antimicrobial resistance mechanism), and Africa. There are at least 150 *bla*_KPC_ gene variants, with the *bla*_KPC-2_ gene being the most prevalent in several countries [141,142].

Infections caused by KPC-producing bacteria have already become endemic in most areas of the world, including Europe (especially Poland and southern Europe countries, such as Italy, Greece, and Spain), Latin America, the US, and Asia [143]. In addition, there have already been sporadic cases of such infections in many additional countries worldwide, including European countries (such as France, Germany, the UK, Ireland, Belgium, the Netherlands, Hungary, Finland, and Sweden), Asia (including South Korea), and Oceania (Australia) [143]. Also, there are reported cases from most remaining countries with a substantial overall record of publications [143]. Finally, there is a paucity of relevant publications from sub-Saharan African countries [143].

In addition to their broad geographic spread, specific KPC-producing lineages have acquired enhanced virulence or additional resistance mechanisms. Recent reports have described hypervirulent *K. pneumoniae* clones (associated with community-acquired invasive disease) that have acquired *bla*_KPC_-harboring plasmids. These plasmids are considered mobile genetic elements (MGEs) and can transfer horizontally between bacterial clones and species. They contain the *bla*_KPC_ genes inside transposons, with the most prevalent of them being the transposon Tn4401, although other elements, such as non-Tn4401, have been reported to contain the genes as well [144]. The plasmids that harbor *bla*_KPC_ genes are categorized as plasmid incompatibility groups, with the two main groups being the IncF plasmids (mainly associated with intra-clonal transfer, or between *K. pneumoniae* and *E. coli*) and the IncN plasmids (transfer between other bacterial species) [145,146]. The hypervirulent *K. pneumoniae* strains are called convergent, as they combine multidrug-resistance with hypervirulence. These cases highlight the possibility of KPC-producing strains causing severe community infections, although their true virulence relative to classical strains is still under investigation. At the same time, strains co-producing KPC together with other carbapenemases (specifically NDM-type metallo-β-lactamases) are increasingly emerging in different parts of the world. The co-production of KPC and NDM in the same isolate has been associated with outbreaks of pan-drug-resistant infections and poses a serious therapeutic challenge [147,148].

Infections due to KPC-producing pathogens usually occur in patients who receive care in the hospital or long-term care facilities. They are more common in patients in intensive care units. KPC-producing pathogens can cause infections in all human systems and organs, including healthcare-associated pneumonia (HAP), such as ventilator-associated pneumonia (VAP), urinary tract infections (UTIs), septicemia, abdominal infections, skin and soft tissue infections (SSTIs), and device-associated infections. In healthcare settings, there is pressure for colonization with KPC-producing pathogens, and opportunities for subsequent cross-infection between patients exist. Invasive medical devices and inadequate infection control measures among healthcare personnel significantly contribute to the transmission and dissemination of infections caused by KPC-producing pathogens in healthcare settings [149]. Adherence to strict infection control practices helps control the dissemination of infections caused by KPC-producing pathogens [150].

Based on the results presented above, the antimicrobial resistance of KPC-producing microorganisms was high in most studied antibiotics, reaching up to 100% (third-generation cephalosporins, cefepime, piperacillin–tazobactam, carbapenems, and fluoroquinolones). Among the antimicrobial agents with potential antimicrobial activity against KPC-producing pathogens (polymyxins, ceftazidime–avibactam, tigecycline, and trimethoprim–sulfamethoxazole), polymyxins may cause nephrotoxicity [151], while tigecycline is not indicated for bacteremia, healthcare-associated pneumonia (due to the presence of adverse events) [152], and urinary tract infections. Ceftazidime–avibactam is a costly drug, especially compared to carbapenems, that has limited availability in low-resource countries [153]. Notably, ceftazidime–avibactam has demonstrated sustained antimicrobial activity against KPC-producing pathogens, with resistance often below 5% in both our analysis and external surveillance data. However, recent evidence highlights the emergence of KPC variants with mutations that impact susceptibility to newer β-lactam/β-lactamase inhibitor (BL/BLI) combinations [154,155]. As described by Hobson et al. [154], specific amino acid substitutions in the Ω-loop region of the KPC enzyme—most notably D179Y—can confer resistance to ceftazidime–avibactam while simultaneously restoring susceptibility to carbapenems. These mutations alter the enzyme’s structure in a way that reduces inhibitor binding but also impairs carbapenem hydrolysis [154,156]. Clinically, this presents a diagnostic and therapeutic challenge, as such variants may not be detected by conventional carbapenemase assays and may respond unpredictably to β-lactam treatment [155,156].

Furthermore, there are limited microbiological and clinical data on the potential use of fosfomycin against KPC-producing pathogens [15]. Thus, the comprehensive evaluation of the published evidence on the susceptibility of KPC-producing bacteria in studies included in our article is of potential clinical interest, particularly in considering the use of various antibiotics for infections caused by such bacteria. In addition, cefiderocol has demonstrated activity against KPC-producing Enterobacterales, with MIC_50_s ranging from 0.15 to 1 mg/L, and could be an option for treating patients with infections caused by KPC-producing bacteria in the absence of other available alternatives [107].

The strength of this review lies in its extensive systematic search of six resources, which evaluates the most recent available data on the epidemiology of KPC-producing pathogens worldwide. Also, the documentation of the implemented search strategy ensures the study’s reproducibility.

However, some limitations need to be addressed. The isolated species refer to all the strains tested for KPC production, and do not represent the exact number of those with positive results via the PCR method. Additionally, not all studies provided relevant data regarding the antimicrobial susceptibility of KPC-producing pathogens; therefore, the results may underrepresent the actual antimicrobial resistance worldwide for these types of bacteria. Last but not least, the included studies were heterogeneous in design and geographic focus. Many regions (particularly lower-income countries) lack adequate surveillance programs for antimicrobial resistance, including for carbapenemase-producing organisms, resulting in potential gaps and biases in the global data. This variability and under-reporting may limit the generalizability of our findings.

## 5. Conclusions

The evaluation of the available and most recent literature shows that KPC-producing pathogens have disseminated worldwide in all continents, with a predominance in Asian countries. Antimicrobial resistance is high in most of the examined antibiotics, with a few exceptions, including polymyxins, ceftazidime–avibactam, tigecycline, and trimethoprim-sulfamethoxazole. However, novel therapeutic options provide hope. New β-lactam/β-lactamase inhibitor combinations, such as meropenem–vaborbactam, imipenem–cilastatin–relebactam, and ceftazidime–avibactam, have demonstrated excellent antimicrobial activity against KPC producers. Additionally, cefiderocol offers an alternative option for treating difficult-to-treat cases. Last but not least, global surveillance and reporting, rigorous infection control practices, and prudent antimicrobial stewardship are crucial to curb the further spread of these highly drug-resistant pathogens [157].

## Figures and Tables

**Table 1:** Detection of Klebsiella pneumoniae carbapenemase (KPC) in Gram-negative bacteria isolated from various regions globally.

First Author, Year [ref.] *	Continent	Country	Isolation Period	Population, n/N (%)	Sources of Isolation (n)	Species (n)	KPC Detection, n/N (%)	Types of KPC
Hassan, 2021 [121]	Africa	Egypt	12/2018–12/2019	Clinical isolates, 154/206 (75) ICU	Wound swabs (77), respiratory secretions (56), blood (37), urine (27), and other (9)	Carbapenem-resistant *A. baumannii*	22/206 (11)	*bla*_KPC_
Odewale, 2023 [122]	Africa	Nigeria	2/2018–8/2019	Clinical isolates	NR	*K. pneumoniae*	17/128 (13)	*bla*_KPC_
Ssekatawa, 2021 [123]	Africa	Uganda	1/2019–12/2019	Clinical isolates	Urine (128), pus swabs (48), blood (23), rectal swabs (16), vaginal swabs (7), tracheal aspirate (3), and sputum (2)	*K. pneumoniae*	18/227 (8)	*bla*_KPC-type_
Fang, 2019 [20]	Asia	China	1/2015–1/2017	Inpatient, 41/47, (87) colonization, 6/47 (13) infection	Respiratory (20), urine (12), blood (6), ascites (3), bile (2), skin (1), and other (3)	CRE; *K. pneumoniae* (35), *E. cloacae* (4), *Citrobacter freundii* (3), *E. coli* (3), and *K. oxytoca* (2)	38/47 (81)	*bla*_KPC-2_
Ge, 2024 [21]	Asia	China	2021–2022	Inpatient	BAL, blood, sputum, and urine	CRKP; *K. pneumoniae*	30/31 (97)	*bla*_KPC-2_
Han, 2020 [22]	Asia	China	1/2016 –12/2018	Inpatient, 498/935 (53) children	Ascites, bile, blood, catheter, drainage, other aseptic body fluids, pus, sputum, and urine	CRE; *K. pneumoniae* (709), *E. coli* (149), *E. cloacae* (36), *Citrobacter freundii* (14), *Serratia marcescens* (8), *E. aerogenes* (7), *K. oxytoca* (7), *Morganella morganii* (3), *Proteus vulgaris* (1), and *Providencia rettgeri* (1)	482/935 (52); 307/935 (70) in adults, 175/935 (35) in children	*bla*_KPC_
Hu, 2020 [23]	Asia	China	2008–2018	Clinical isolates	NR	CRKP; *K. pneumoniae*	504/534 (94)	*bla*_KPC-2_
Jin, 2022 [24]	Asia	China	1/2019–12/2021	Inpatient, children	Sputum, urine, blood, catheter, BAL, gastric fluid, and others	*K. pneumoniae*	21/34 (62)	*bla*_KPC-2_
Jing, 2022 [25]	Asia	China	07/2019–10/2019	NR	Respiratory tract (129), blood (45), urine (17), wounds (8), ascitic fluid (6), pleural effusion (5), cerebrospinal fluid (4), and other (24)	*K. pneumoniae* (184), *E. cloacae* (11), *K. oxytoca* (6), *Citrobacter freundii* (5), *K. aerogenes* (2), and *Serratia marcescens* (1)	175/238 (74)	*bla*_KPC-2_
Kang, 2020 [26]	Asia	China	1/2016–12/2016	ICU	Blood, CSF, lower respiratory tract, urine, and wounds	CRKP; *K. pneumoniae*	120/128 (94)	*bla*_KPC-2_
Li, 2021 [27]	Asia	China	2015–2018	NR	Lower respiratory tract (249), urine (33), secretion (25), blood (14), catheter (13), puncture fluid (7), pleural fluid (7), drainage fluid (4), ascitic fluid (3), cerebral spinal fluid (2), pus (2), and bile (1)	*K. pneumoniae* (350), *E. coli* (26), *E. cloacae* (15), and other (8)	119/399 (30)	*bla*_KPC-2_
Liang, 2024 [28]	Asia	China	1/2013–12/2022	Inpatient	Bloodstream infections	CRE; *K. pneumoniae*, *E. coli*, *E. cloacae*, and *K. oxytoca*, *Salmonella* spp.	8/35 (23)	*bla*_KPC_
Liao, 2023 [29]	Asia	China	01/2018–02/2021	Clinical isolates	Sputum (17), bronchoalveolar lavage fluid (8), urine (7), blood (3), secretion (3), and others (4)	*K. pneumoniae*	35/42 (83)	*bla*_KPC-2_
Liu, 2021 [30]	Asia	China	2004–2018	Clinical isolates	NR	*E. cloacae* complex	14/113 (12)	*bla*_KPC-2_
Ma, 2024 [31]	Asia	China	1/2021–12/2021	Inpatient, children	Sputum (63), blood (11), urine (10), BAL (9), ascites (5), CSF (3), joint fluid (1), hydrothorax (1), and other (2)	CRKP; *K. pneumoniae*	93/108 (86)	*bla*_KPC_
Peng, 2022 [32]	Asia	China	1/2015–12/2018	Inpatient, 36/89 (40) ICU	Sputum (86), urine (31), blood (13), lung tissue (9), and bile (7)	CRE; *K. pneumoniae* (89), *E. coli* (28), *E. cloacae* (20), *Citrobacter freundii* (5), *K. aerogenes* (3), and *Cronobacter sakazakii* (1)	73/146 (50)	*bla*_KPC-2_
Shen, 2023 [33]	Asia	China	01/2018–12/2020	Clinical isolates	NR	*K. pneumoniae*	23/94 (24)	*bla*_KPC_
Shi, 2024 [34]	Asia	China	2018–2019	Clinical isolates	NR	CRKP; *K. pneumoniae* (708)	563/708 (80)	*bla*_KPC-2_
Tian, 2018 [35]	Asia	China	1/2016–12/2017	Inpatient, children	NR	CRKP; *K. pneumoniae*	30/169 (18)	*bla*_KPC-2_
Tian, 2020 [36]	Asia	China	2002–2017	Clinical isolates	Urine (18), blood (16), drainage (11), and other (13)	*E. coli*	13/58 (22)	*bla*_KPC-2_
Wang, 2018 [37]	Asia	China	1/2012 –12/2016	Clinical isolates	Respiratory tract (858), blood (304), urine (303), abdominal fluid (117), and other (219)	CRE; *K. pneumoniae* (1201), *E. coli* (282), *E. cloacae* (179), *Citrobacter freundii* (44), *K. oxytoca* (29), *Serratia marcescens* (28), *E. aerogenes* (24), *Raoultella ornithinolytica* (6), *Citrobacter braakii* (3), *Citrobacter koseri* (3), and *Raoultella planticola* (2)	961/1801 (53)	*bla*_KPC-2_
Wang, 2020 [39]	Asia	China	10/2016–3/2019	Inpatient	Sputum (12), drainage fluid (4), blood (3), wound (2), urine/urinary catheter (7), and bronchial perfusate (1)	CRKP; *K. pneumoniae*	12/30 (40)	*bla*_KPC_
Wang, 2022 [38]	Asia	China	3/2018–6/2018	Inpatient	Sputum (18), blood (16), pus (6), ascites (2), CSF (1), bile (1), and secretions (1)	CRKP; *K. pneumoniae*	42/45 (93)	*bla*_KPC-2_
Wei, 2022 [40]	Asia	China	1/2020–12/2020	ICU	Blood, pus, puncture fluid, respiratory tract, and urine	CRKP; *K. pneumoniae*	79/80 (99)	*bla*_KPC-2_
Wu, 2024 [41]	Asia	China	2021	Inpatient	Blood, CNS, gastrointestinal tract, genital tract, peritoneum, respiratory tract, skin/soft tissue, and urinary tract	Carbapenem-resistant: *A. baumannii*, *Enterobacterales*, *E. coli*, *K. pneumoniae*, and *P. aeruginosa*	203/1257 (16)	*bla*_KPC_
Yan, 2021 [42]	Asia	China	2018–2019	Clinical isolates	Sputum (142), blood (82), urine (21), ascites (11), wound (9), CSF (5), and other (35)	CRE; *K. pneumoniae*, *E. coli*, *E. cloacae*, *Citrobacter freundii*, *K. oxytoca*, *Providencia rettgeri*, *K. aerogenes*, and *Serratia marcescens*	215/305 (70)	*bla*_KPC_
Yang, 2013 [43]	Asia	China	2/2009–11/2011	Inpatient, ICU	Sputum (25), blood (8), urine (7), venous cannula (3), drainage fluid (3), and bile (2)	*K. pneumoniae*	2/2009–11/2011: 48/1636 (3) 12/2012–6/2012: 41/351 (12)	*bla*_KPC-2_
Zhang, 2021 [44]	Asia	China	4/2018–7/2019	Inpatient, 102/133 (77) ICU	Sputum (81), bile (12), urine (12), blood (8), and CSF and BAL (7)	CRKP; *K. pneumoniae*	133/133 (100)	*bla*_KPC-2_
Patil, 2023 [63]	Asia	India	1/2020–12/2021	Inpatients, outpatient	Blood, urine, wound, sputum, and CSF	*K. pneumoniae*	13/60 (22)	*bla*_KPC_
Darabi, 2019 [64]	Asia	Iran	12/2013–9/2016	Inpatient, 107/182 (59) outpatient	Urine (137) and sputum (26), wounds (10), blood (8), and stool (1)	*K. pneumoniae*	7/182 (4)	*bla*_KPC_
Haji, 2021 [137]	Asia	Iraq	2019–2020	Clinical isolates, inpatient and outpatient	Urine, sputum, swabs, and blood	*E. coli*, *A. baumannii*, and *Achromobacter dentrificans*	4/53 (7)	*bla*_KPC_
Adler, 2011 [46]	Asia	Israel	8/2008–4/2009	Inpatient	Rectal swabs	CRE; *K. pneumoniae*, *K. oxytoca*, and *E. aerogenes*	32/33 (97)	*bla*_KPC_
Adler, 2015 [45]	Asia	Israel	1/2009–6/2012	Inpatient	Surveillance cultures (76) and blood (12)	*E. coli*	88/88 (100)	*bla*_KPC-2_, *bla*_KPC-3_
Ben-David, 2015 [47]	Asia	Israel	1/2006–5/2007	Inpatient	Blood and urine	CRKP; *K. pneumoniae*	120/120 (100)	*bla*_KPC-3_
Hussein, 2022 [48]	Asia	Israel	1/2005–12/2020	Inpatient	Rectal swabs	CPE; *Citrobacter* spp., *E. coli*, *E. cloacae*, *K. oxytoca*, *K. pneumoniae*, *Morganella* spp., *Proteus* spp., *Providencia* spp., and *Raoultella* spp.	2014: 89/95 (94) 2015: 100/109 (92) 2016: 51/59 (84) 2017: 58/65 (88) 2018: 58/88 (66) 2019: 71/141 (51) 2020: 75/134 (56) 2014–2020: 502/691 (73)	*bla*_KPC_
Manandhar, 2020 [49]	Asia	Nepal	6/2012–12/2018	Inpatient, 295/2153 (14) children	NR	*E. coli* (719), *Klebsiella* spp. (532), and *Enterobacter* spp. (520), and *A. baumannii* (383)	*E. coli*: 0/719 (0) *Klebsiella* spp.: 22/532 (4) *Enterobacter*: 201/337 (60) ^a^ *A. baumannii*: not tested	*bla*_KPC_
Sah, 2021 [50]	Asia	Nepal	1/2016–12/2016	Inpatient, ICU, children, neonates	Blood	*A. baumannii* complex, *E. coli*, *E*, *aerogenes*, and *K. pneumoniae*	8/50 (16)	*bla*_KPC_
Takahashi, 2021 [51]	Asia	Nepal	10/2018–1/2020	Hospital samples	NR	*P. aeruginosa*	4/43	*bla*_KPC-2_
AlAmri, 2019 [62]	Asia	Saudi Arabia	9/2017–5/2018	Clinical isolates	Respiratory tract (41), wound swabs (18), rectal swabs (13), blood (10), urine (6), and other (15)	*A. baumannii*	103/103 (100)	*bla*_KPC-like_
Ling, 2015 [52]	Asia	Singapore	1/2011–12/2013	Inpatient	Stool, urine, wound, blood, sputum, bile, catheter, peritoneal fluid, mephrostomy fluid, tracheal aspirate, and ulcer	CRE; *K. pneumoniae*, *E. coli*, and *E. cloacae* complex	107/268 (40)	*bla*_KPC_
Teo, 2014 [53]	Asia	Singapore	9/2010–5/2013	Clinical isolates	NR	CRE; *E. coli*, *Klebsiella* spp., *Citrobacter* spp., and *Enterobacter* spp.	31/400 (8)	*bla*_KPC-type_
Teo, 2022 [54]	Asia	Singapore	2009–2020	Inpatient	NR	CRKP; *K. pneumoniae sensu stricto* (500), *K. quasipneumoniae* subsp. *similipneumoniae* (55), *K. quasipneumoniae* subsp. *quasipneumoniae* (55), and *K. variicola* subsp. *variicola* (9)	235/575 (41)	*bla*_KPC_
Lim, 2024 [1]	Asia	South Korea	2018–2022	Clinical isolates	NR	CPE; *K. pneumoniae*, *E. coli*, *Enterobacter* spp., *Citrobacter freundii*, *K. oxytoca*, *Serratia marcescens*, *Citrobacter koseri*, *Raoultella ornitholytica*, *Providencia rettgeri*, *Proteus* spp., and *Morganella morganii*	47,313/63,513 (74)	*bla*_KPC_
Yoo, 2023 [59]	Asia	South Korea	01/2016–12/2021	Inpatients from ICU	NR	*K. pneumoniae* (253), *E. cloacae complex* (44), *E. coli* (15), and others (15)	164/327 (50)	*bla*_KPC_
Chiu, 2013 [55]	Asia	Taiwan	2010–2012	Clinical isolates	NR	CRKP; *K. pneumoniae*	41/347 (12)	*bla*_KPC-2_
Huang, 2023 [56]	Asia	Taiwan	2013–2021	NR	Urine (68), blood (50), sputum (37), skin/pus/wound (14), body fluids (5), and catheter tip (1)	CPE; *K. pneumoniae* (79), *E. coli* (56), *E. cloacae* complex (44), *Citrobacter freundii* (9), *K. oxytoca* (6), and *K. aerogenes* (1)	38/195 (19)	*bla*_KPC_
Lee, 2021 [57]	Asia	Taiwan	2017–2020	Inpatient	NR	CRE; *K. pneumoniae* (175), *E. coli* (26)	2017: 64/83 (77) 2018: 27/36 (75) 2019: 37/43 (85) 2020: 32/39 (82) 2017–2020: 69/201 (34)	*bla*_KPC_
Falco, 2016 [65]	Asia	Venezuela	4/2014–7/2014	Inpatient, children, 2/19 (11) ICU	Blood (15), bronchial secretion (2) catheter (1), and lesion secretion (1)	CRKP; *K. pneumoniae*	19/19 (100)	*bla*_KPC-2_
Berglund, 2019 [60]	Asia	Vietnam	2/2015–9/2015	Inpatient	Tracheal fluid, nasopharynx, blood, and other	*K. pneumoniae*	57/57 (100)	*bla*_KPC-2_
Linh, 2021 [61]	Asia	Vietnam	2010–2015	Inpatient	Bronchial fluid (92), blood (18), sputum (6), urine (4), pleural fluid (1), abdominal fluid (1)	CRE; *K. pneumoniae* (305), *E. coli* (186), *Klebsiella* spp. (54), *Enterobacter* spp. (29), and *Citrobacter* spp. (25)	122/599 (20) ^b^	*bla*_KPC-2_, *bla*_KPC-12_
Miriagou, 2010 [13]	Asia/Europe	Russia	1/2020–12/2021	Inpatient	Blood, cerebrospinal fluid, urine, BAL, endotracheal aspirate, biopsies, and wound swabs	*K. pneumoniae* (2503) and *E. coli* (2055)	410/4558 (9)	*bla*_KPC-3_
Yürek, 2023 [66]	Asia/Europe	Turkey	12/2020–3/2021	Inpatient, outpatient	Urine (27), blood (6), respiratory tract (6), abscess or wound (3), and other (3)	*K. pneumoniae*	5/35 (14)	*bla*_KPC_ ^c^
David, 2019 [93]	Europe	31 countries ^d^	11/2013–4/2014	NR	NR	*K. pneumoniae* spp.	311/1649 (18)	*bla*_KPC-2_, *bla*_KPC-3_, *bla*_KPC-12_
Grundmann, 2017 [95]	Europe	Albania, Austria, Belgium, Bulgaria, Croatia, Cyprus, Czech Republic, Denmark, Estonia, Finland, France, Germany, Greece, Hungary, Iceland, Ireland, Israel, Italy, Kosovo, Latvia, Lithuania, Luxembourg, Malta, Montenegro, Norway, Poland, Portugal, Romania, Serbia, Slovakia, Slovenia, Spain, Sweden, Macedonia, Turkey, the UK—England and Northern Ireland, and the UK—Scotland	11/2013–4/2014	Inpatient	All clinical specimens were accepted, except for stool and surveillance screening samples	*K. pneumoniae* (850), *E. coli* (77)	393/927 (42)	*bla_KPC_*
Hoenigl, 2012 [81]	Europe	Austria	10/2010–2/2011	Inpatient	NR	*K. oxytoca*	31/31 (100)	*bla_KPC_*
Kazmierczak, 2020 [94]	Europe	Austria, Belgium, Denmark, France, Germany, the Netherlands, Sweden, the UK, Greece, Italy, Portugal, Spain, Turkey, the Czech Republic, Hungary, Poland, Romania, and Russia	2013–2017	NR	Lower respiratory tract, skin and soft tissue, urinary tract, intra-abdominal, bloodstream, or other infections	CRE; *K. pneumoniae*, *E. cloacae*, and *E. coli*	570/1231	*bla*_KPC_
De Laveleye, 2017 [82]	Europe	Belgium	1/2013–12/2014	NR	NR	*E. coli* and *K. pneumoniae*	2013: 13/35 (37) 2014: 25/35 (71)	*bla*_KPC_
Dobreva, 2022 [83]	Europe	Bulgaria	2014–2018	NR	Urine (4), blood (3), wound (2), cerebrospinal fluid (1), tracheobronchial aspirate (1), and rectal swab (1)	*K. pneumoniae*	12/12 (100)	*bla_KPC-2_*
Hammerum, 2020 [84]	Europe	Denmark	1/2014–6/2018	Clinical isolates	NR	*K. pneumoniae*, *Klebsiella quasipneumoniae*, and *Klebsiella variicola*	8/103 (8)	*bla*_KPC-2_, *bla*_KPC-3_
Räisänen, 2020 [85]	Europe	Finland	2012–2018	Clinical isolates	Urine, wound swabs, blood, respiratory tract	CPE; *K. pneumoniae*, *E. coli*, and *Citrobacter freundii*, *E. cloacae*	71/231 (31)	*bla*_KPC-like_
Carbonne, 2010 [86]	Europe	France	9/2009–10/2009	Inpatient, 4/13 (31) infection, 9/13 (69) colonization ^c^	Bile, blood, bronchial aspirate, and rectal swab	*K. pneumoniae*	13/295 (4)	*bla*_KPC-2_
Bonnin, 2024 [92]	Europe	Germany, Belgium, England, Austria, Netherlands, Poland, and Czech Republic	1/2013–3/2021	NR	NR	Carbapenemase-producing *Morganella morganii*	26/247 (11)	*bla*_KPC-2_
Kontopidou, 2014 [68]	Europe	Greece	9/2009–6/2010	ICU	NR	CPKP; *K. pneumoniae*	52/82 (63)	*bla*_KPC_
Pournaras, 2013 [69]	Europe	Greece	9/2010–2/2012	Inpatient	Rectal swab	CPE; *K. pneumoniae*, *E. coli*	56/97 (58)	*bla*_KPC_
Sorovou, 2023 [70]	Europe	Greece	1/2019–12/2022	Inpatient, 26/212 (12) ICU	Urine (154), rectal swab (12), BAL (11), wound swabs (8), sputum (7), central venous catheter (5), and other (15)	*K. pneumoniae*	2019: 10/51 (20) 2020: 8/42 (19) 2021: 9/54 (17) 2022: 38/65 (58) 2019–2022: 65/212 (31)	*bla*_KPC_
Zarras, 2022 [71]	Europe	Greece	3/2018–3/2021	ICU, 34/150 (23) pediatric/neonatal	Blood, bronchial secretions, rectal swabs, trauma, and urine ^e^	CRKP; *K. pneumoniae*	56/150 (37), 24/34 (71) in children and neonates	*bla*_KPC_
Budia-Silva, 2024 [11]	Europe	Greece, Italy, Romania, Serbia, Spain, and Turkey	2016–2018	Inpatient	Blood (334), urine (213), and other (140)	CRKP; *K. pneumoniae*	384/683 (56)	*bla*_KPC-2_, *bla*_KPC-3_, *bla*_KPC-36_
Buzgó, 2025 [79]	Europe	Hungary	3/2021–4/2023	Inpatient	NR	*K. pneumoniae*	53/420 (13)	*bla_KPC-2_*, *bla_KPC-3_*
Toth, 2010 [80]	Europe	Hungary	9/2008–4/2009	Inpatient	Wound (2), lower respiratory tract (2), upper respiratory tract (2), stool (2), and central venous catheter (1)	*K. pneumoniae*	9/9 (100)	*bla_KPC-2_*
Morris, 2012 [87]	Europe	Ireland	2011	Inpatient	Blood, central venous catheter, rectal swab, and urine	CRKP; *K. pneumoniae*	19/19 (100)	*bla*_KPC-2_
Agodi, 2011 [72]	Europe	Italy	3/2009–5/2009	ICU	Sputum (7), blood (6), urine (5), tonsillar swab (3), catheter tip (2), and peritoneal fluid (1)	CRKP; *K. pneumoniae*	24/24 (100)	*bla*_KPC-3_
Orena, 2024 [73]	Europe	Italy	1/2024–6/2024	Inpatient	Rectal swabs	CRE; *E. coli*, *E. cloacae*, *K. oxytoca*, and *K. pneumoniae*	30/118 (25)	*bla*_KPC_
Piazza, 2024 [74]	Europe	Italy	10/2021–3/2022	Inpatient, infection, colonization	Blood (14), rectal swabs (3), respiratory tract (3), urine (1)	Ceftazidime-resistant; *K. pneumoniae*	21/21 (100)	*bla*_KPC_
Santino, 2013 [75]	Europe	Italy	1/2012–6/2012	Clinical isolates	Urine (5), wound (3), sputum (3), BAL (1), skin swab (l), blood (1), and pharyngeal swab (1)	*K. pneumoniae*	15/15 (100)	*bla*_KPC-3_
Jamin, 2024 [88]	Europe	Netherlands	2011–2023	Clinical isolates	NR	*K. pneumoniae*	107/2985 (4)	*bla*_KPC-3_
Samuelsen, 2017 [89]	Europe	Norway	2007–2014	Inpatient, traveling abroad	Urine and fecal screening	CPE; *K. pneumoniae* and *E. cloacae*	20/59 (34)	*bla*_KPC-2_, *bla*_KPC-3_
Guzek, 2019 [76]	Europe	Poland	1/2009–12/2016	Inpatients	Urine (46), cloacal swabs for fecal carriage (43), blood (11), wounds (9), bronchial tree aspirates collected via an endotracheal tube (8), abscesses (3), and fluid collected from the abdominal cavity (2)	*K. pneumoniae*, *E. coli*, and *C. freundii*	73/122 (60)	*bla_KPC_*
Kuch, 2020 [77]	Europe	Poland	1/2000–1/2017	NR	Urine, blood, and other clinical specimens (bronchial secretions, cerebrospinal fluid, peritoneal fluid, pleural fluid, pus, skin lesions, sputum, and wounds)	*K. pneumoniae*, *K. oxytoca*, *P. mirabilis*, *P. penneri*, *P. vulgaris*, *C. freundii*, *C. braakii*, *E. cloacae*, *E. aerogenes*, *E. amnigenus*, *S. marcescens*, *M. morganii*, and *P. rettgeri*	40/400 (10)	*bla_KPC_*
Mrowiec, 2019 [78]	Europe	Poland	2008–2015	Inpatient	Children: stool (69) and perianal swabs (12); adults: respiratory system (39), urine (23), and blood (10)	*K. pneumoniae*	14/170 (8)	*bla_KPC_*
Baicus, 2018 [90]	Europe	Romania	2016	Inpatient, ICU	Lower respiratory tract (20), urine (30), blood (4), peritoneum (1), wound (5), and sputum (1)	*K. pneumoniae*	6/20 (30)	*bla_KPC_*
Gracia-Ahufinger, 2023 [91]	Europe	Spain	1/2014–12/2018	Inpatient	NR	CPE; *K. pneumoniae*, *E. cloacae* complex, *E. coli*, and *K. aerogenes*	313/2280 (14)	*bla*_KPC_
Kazmierczak, 2016 [126]	Global	40 countries	2012–2014	Inpatient, 195/586 (33) ICU ^f^	Respiratory tract (189), urinary tract (137), skin/soft tissue (132), and wounds (71) ^f^	CRE; *Enterobacterales* (38,266) and *P. aeruginosa* (8010)	586/46,276 (1)	*bla*_KPC-2_, *bla*_KPC-3_, *bla*_KPC-9_, *bla*_KPC-12_, *bla*_KPC-18_
Nobrega, 2023 [127]	Global	56 countries	2015–2017	NR	NR	*Citrobacter freundii* (51), *Citrobacter portucalensis* (20), *Citrobacter koseri* (10), *Citrobacter farmeri* (3), *Citrobacter amalonaticus* (1), and *Citrobacter braakii* (1)	29/91 (32)	*bla*_KPC-2_, *bla*_KPC-3_
Estabrook, 2023 [125]	Global	NR	2018–2019	NR	NR	Meropenem-resistant; *K. pneumoniae*, *E. cloacae*, *E. coli*, *Providencia* spp., *Serratia marcescens*, *Klebsiella* spp., *Citrobacter* spp., *Enterobacter* spp., *Proteus* spp., and *Morganella morganii*	568/2228 (26)	*bla*_KPC-2_, *bla*_KPC-3_, *bla*_KPC-4_, *bla*_KPC-6_, *bla*_KPC-31_, *bla*_KPC-46_, *bla*_KPC-66_
Castanheira, 2012 [124]	Global	NR ^g^	1999–2008	Inpatient	NR	CPE; *E. coli* and *Klebsiella* spp.	56/69 (81)	*bla*_KPC_
Issac, 2023 [108]	North America	Canada	2013–2020	Inpatient	Urine (917), axilla/groin/rectum (343), wounds (197), respiratory tract (163), blood (84), other (91), and unknown (10)	CRE; *K. pneumoniae* (542), *E. cloacae* (508), *E. coli* (238), others (512), and unknown (5)	406/1805 (22)	*bla*_KPC_
Mataseje, 2016 [109]	North America	Canada	1/2010–12/2014	Inpatient, outpatient	Stool or rectal swab, urine, sputum, blood, skin or soft tissue, surgical site, and other	CPE; *K. pneumoniae*, *Enterobacter* spp., *Serratia* spp., *Citrobacter* spp., *K. oxytoca*, *Morganella morganii*, *Providencia rettgeri*, *Pantoea* spp., and *Kluyvera* spp.	169/261 (65)	*bla*_KPC-2_, *bla*_KPC-3_, *bla*_KPC-4_
Bradford, 2004 [96]	North America	USA	11/1997–7/2001	Inpatient	Urine (9), sputum (8), blood (1), and miscellaneous sources (3) ^h^	CPE; *K. pneumoniae* (18) and *K. oxytoca* (1)	19/19 (100)	*bla*_KPC-2_
Castanheira, 2017 [97]	North America	USA	2012–2015	Inpatient	Pneumonia, urinary tract infections, skin/soft tissue infections, bloodstream infections, and intra-abdominal infections	CRE; *K. pneumoniae*, *E. cloacae* species complex, *E. coli*, *K. oxytoca*, *Serratia marcescens*, *Citrobacter freundii*, and *E. aerogenes*	456/525 (87)	*bla*_KPC-2_, *bla*_KPC-3_, *bla*_KPC-4_, *bla*_KPC-17_ ^i^
Endimiani, 2009 [98]	North America	USA	3/2008–4/2008	Inpatient	Blood, respiratory tract, and urine	*K. pneumoniae*	10/241 (4) (total), 10/10 (100) (CRKP)	*bla*_KPC-2_, *bla*_KPC-3_
Fitzpatrick, 2022 [99]	North America	USA	1/2013–12/2018	Inpatient, outpatient	Urine (1163), respiratory tract (235), blood (189), rectal (28), and other (290)	Carbapenemase-producing CRE; *E. coli*, *K. pneumoniae*, *K. oxytoca*, and *Enterobacter* spp.	914/1047 (87)	*bla*_KPC_
Gomez-Simmonds, 2021 [100]	North America	USA	3/2020–4/2020	Inpatient	Respiratory tract (14), blood (5), and urine (1)	CPE; *K. pneumoniae*	27/31 (87)	*bla*_KPC-2_, *bla*_KPC-3_
Jacobs, 2019 [107]	North America	USA	NR	NR	NR	*K. pneumoniae* (794), *E. coli* (35), *Enterobacter* spp. (4), and *Citrobacter freundii* (1)	737/834 (88)	*bla*_KPC-2_, *bla*_KPC-3_, *bla*_KPC-4_, *bla*_KPC-4-like_
Kaiser, 2013 [101]	North America	USA	2007–2009	Inpatient	Blood, skin and soft tissue, respiratory tract, urinary tract	*K. pneumoniae*	113/2049 (6)	*bla*_KPC_
Karlsson, 2022 [106]	North America	USA	2011–2015	Inpatient	Urine (349), blood (58), and other normally sterile sites (14)	CPE; *K. pneumoniae* (265), *E. cloacae complex* (77), *E. coli* (50), *K. aerogenes* (26), and *K. oxytoca* (3)	299/307 (97)	*bla_KPC_*
Logan, 2019 [102]	North America	USA	1/2008–12/2014	Inpatient, children	Urine (9), blood (9), respiratory tract (8), and other (10)	*K. pneumoniae*, *E. coli*, and *E. cloacae*	18/36 (50)	*bla*_KPC_
Precit, 2020 [103]	North America	USA	10/2010–12/2017	Clinical isolates	Urine (45), bronchial wash or sputum (7), wound (6), blood (3), stool or rectal swab (5), and other (7)	*E. coli* and *Klebsiella* spp.	35/74 (47)	*bla*_KPC_
Shortridge, 2023 [104]	North America	USA	2016–2020	Inpatient	Bloodstream infections, pneumonia, and urinary tract infections	CRE; *E. coli*, *K. pneumoniae*, and *E. cloacae*	184/222 (83)	*bla*_KPC-2_, *bla*_KPC-3_, *bla*_KPC-4_, *bla*_KPC-6_
van Duin, 2020 [105]	North America	USA	4/2016–8/2017	Inpatient	Urine (404), respiratory tract (268), blood (130), wound (130), intra-abdominal (58), and other (50)	*K. pneumoniae*, *Enterobacter* spp., *E. coli*, non-*K. pneumoniae Klebsiella* spp., and other	573/1040 (55)	*bla*_KPC-2_*, bla*_KPC-3_, *bla*_KPC-4_, *bla*_KPC-6_, *bla*_KPC-8_, *bla*_KPC-18_
Echegorry, 2024 [118]	South America	Argentina	11/2021	Clinical isolates	Urine, blood, respiratory tract, abdominal tract, and others	*K. pneumoniae* (628), *Morganellaceae* (57), *Enterobacter cloacae* complex (51), *E. coli* (38), and other (47) ^j^	327/821 (40)	*bla*_KPC_
Borghi, 2023 [110]	South America	Brazil	4/2015–11/2015	Inpatient	Blood, bone fragment, catheter, nasal swab, rectal swab, secretions, tracheal aspirate, urine, and wound swab	CRKP; *K. pneumoniae*	40/40 (100)	*bla*_KPC_
Campos, 2017 [111]	South America	Brazil	3/2013–3/2014	ICU	Urine, swab surveillance, tracheal aspirate, blood, surgical wound, catheter tip, biological fluids, CSF, and skin biopsy	*K. pneumoniae*	149/165 (90)	*bla*_KPC_
Fochat, 2024 [112]	South America	Brazil	1/2020–8/2023	Inpatient, ICU	NR	CRE; *K. pneumoniae*, *Serratia marcescens*, *E. cloacae*, *K. aerogenes*, and *E. coli*	9/46 (20)	*bla*_KPC_
Kiffer, 2023 [113]	South America	Brazil	2015–2022	Inpatient	NR	*Enterobacterales*, *P. aeruginosa*, and *A. baumannii*	*Enterobacterales*: 41,282/60,205 (68) *P. aeruginosa*: 1065/12,625 (8) *A. baumannii*: 52/10,452 (0.5)	*bla*_KPC_
Tavares, 2015 [114]	South America	Brazil	1/2009–12/2011	NR	Surveillance swabs (26), urine (13), respiratory tract (12), skin and soft tissue (10), blood (5), other (10), and NR (7) ^k^	CRE; *E. aerogenes* (121), *E. coli* (104), *E. cloacae* (100), *Serratia marcescens* (19), *Providencia stuartii* (11), *Pantoea agglomerans* (10), *Citrobacter freundii* (8), *K. oxytoca* (9), and *Morganella morganii* (5)	83/387 (21)	*bla*_KPC-2_
Tolentino, 2019 [115]	South America	Brazil	2011–2014	Inpatient, 48/48 (100)	NR	*K. pneumoniae*	48/48 (100)	*bla*_KPC-2_
Quesille-Villalobos, 2025 [119]	South America	Chile	1/2019–12/2022	Inpatient	Biopsies, blood, bone tissue, sterile fluids, and other	CPE; *K. pneumoniae*, *E. cloacae complex*, *E. coli*, *Citrobacter* spp., and *K. oxytoca*	2019: 8/12 (67) 2020: 35/60 (58) 2021: 69/168 (41) 2022: 87/190 (46) 2019–2020: 199/430 (46)	*bla*_KPC-2_, *bla*_KPC-3_
Ibáñez-Prada, 2024 [116]	South America	Colombia	7/2017–7/2021	Inpatient, 189/248 (76) ICU	NR	CRE; *K. pneumoniae*, *Enterobacter hormaechei*, *Klebsiella variicola* subsp. *variicola*, *Providencia rettgeri*, and *P. aeruginosa*	171/228 (75)	*bla*_KPC-2_, *bla*_KPC-3_
Ocampo, 2016 [117]	South America	Colombia	6/2012–6/2014	Inpatient, 28/193 (15) children, 55/193 (29) ICU	NR	*K. pneumoniae*	166/193 (86)	*bla*_KPC_
Soria-Segarra, 2024 [120]	South America	Ecuador	1/2022–5/2022	Clinical isolates	NR	CPE; *K. pneumoniae*, *K. aerogenes*, *E. cloacae*, *E. coli*, *A. baumannii*, and *P. aeruginosa*	52/60 (87)	*bla*_KPC_
Lascols, 2012 [135]	Various; Africa, Asia, Europe, Latin America, the Middle East, North America, South Pacific	NR	2008–2009	Clinical isolates	Intra-abdominal infections	*E. coli*, *K. oxytoca*, *K. pneumoniae*, and *Proteus mirabilis*	28/1093 (3)	*bla*_KPC-2_, *bla*_KPC-3_, *bla*_KPC-11_
Wise, 2023 [136]	Various; Africa/Middle East, Asia, Eurasia, Latin America	27 countries ^l^	2019–2021	Inpatient, 6632/24,937 (27) ICU	Urinary tract (6408), bloodstream (5921), lower respiratory tract (4907), skin/soft tissue (4400), intra-abdominal (3263), and other (38)	*Enterobacterales*	705/7446 ^m^ (9)	*bla*_KPC_
Hawser, 2009 [131]	Various; America, Asia, Europe	Israel, Puerto Rico, Colombia, and Greece	2005–2008	Clinical isolates	NR	NR	26/86 (30)	*bla*_KPC_
Doyle, 2012 [129]	Various; Asia, Europe, Latin America, Middle East, North America, the South Pacific	NR	2008–2009	Clinical isolates	NR	CPE; *Klebsiella* spp., *E. coli*, *Citrobacter freundii*, and *Enterobacter* spp.	49/142 (35)	*bla*_KPC_
Karlowsky, 2022 [132]	Various; Asia, Europe, Latin America, North America	52 countries	2018–2020	NR	Urinary tract (1210), respiratory tract (998), blood (823), intra-abdominal (409), and skin/soft tissue (449)	*Enterobacterales*	230/1265 (18)	*bla*_KPC_
Gales, 2023 [130]	Various; Asia/Pacific, Europe, Latin America, Middle East/Africa, North America	NR ^n^	2017–2019	Inpatient	Blood, intra-abdominal, other (nervous system, reproductive system, head, ears, eyes, nose and throat), respiratory tract, skin/musculoskeletal, urinary tract, and instruments	*E. coli* (20,047), *P. aeruginosa* (20,643), *K. pneumoniae* (17,229), and *E. cloacae* (6866)	830/64,785 (1)	*bla*_KPC-2_, *bla*_KPC-3_
Castanheira, 2019 [128]	Various; Asia/Pacific, Europe, Latin America, North America	42 countries	2007–2016	NR	NR	CRE; *K. pneumoniae* (997), *E. cloacae* species complex (69), *E. coli* (52), *Serratia marcescens* (44), *K. oxytoca* (30), *K. aerogenes* (27), *Citrobacter freundii* species complex (9), *Proteus mirabilis* (6), *Enterobacter* spp. (4), *Raoultella ornithinolytica* (3), *Pluralibacter gergoviae* (2), *Providencia stuartii* (2), *Raoultella planticola* (2), *Serratia* spp. (2), *Hafnia alvei* (1), *Morganella morganii* (1), *Pantoea agglomerans* (1), and *Raoultella* spp. (1)	2007–2009: 186/1298 (50) 2014–2016: 501/1298 (54)	*bla*_KPC-2_, *bla*_KPC-3_, *bla*_KPC-4_, *bla*_KPC-6_, *bla*_KPC-12_, *bla*_KPC-17_, *bla*_KPC-20_, *bla*_KPC-like_ ^o^
Kazmierczak, 2021 [133]	Various; Asia/South Pacific, Europe, Latin America, Middle East/Africa, North America	34 countries ^p^	2012–2017	Inpatient	Lower respiratory tract (778), urinary tract (631), skin and soft tissue (581), intra-abdominal (408), bloodstream (266), and other (2)	Meropenem non-susceptible; *K. pneumoniae* (2046), *E. cloacae* (177), *E. coli* (136), *Klebsiella* spp. (101), *Citrobacter* spp. (79), *Proteeae* (70), *Serratia marcescens* (31), *Enterobacter* spp. (18), and *Raoultella* spp. (8)	1263/2666 (47)	*bla*_KPC-2_, *bla*_KPC-3_, *bla*_KPC-9_, *bla*_KPC-12_, *bla*_KPC-17_, *bla*_KPC-18_, *bla*_KPC-29_, *bla*_KPC-30_
Kazmierczak, 2019 [134]	Various; Europe, North America	Canada, Czech Republic, France, Germany, Greece, Hungary, Italy, Russia, Spain, Sweden, Turkey, and the United Kingdom	2014	Inpatient	NR	*A. baumannii*, *E. cloacae*, *K. oxytoca*, *K. pneumoniae*, *P. aeruginosa*, and *Serratia marcescens*	75/1272 (6)	*bla*_KPC_

Abbreviations: *A. baumannii*, *Acinetobacter baumannii*; BAL, bronchoalveolar lavage; CPE, carbapenemase-producing *Enterobacterales*; CRE, carbapenem-resistant *Enterobacterales*; CNS, central nervous system; CRKP, carbapenem-resistant *Klebsiella pneumoniae*; CSF, cerebrospinal fluid; *E. aerogenes*, *Enterobacter aerogenes*; *Enterobacter cloacae*, *E. cloacae*; *E. coli*, *Escherichia coli*; *K. aerogenes*, *Klebsiella aerogenes*; *K. oxytoca*, *Klebsiella oxytoca*; *K. pneumoniae*, *Klebsiella pneumoniae*; *K. pneumoniae*; CRPA, *carbapenem-resistant P. aeruginosa*. * Studies are presented in alphabetical order first by continent and then by country. If multiple counties are included in a study, then the sorting is based on the first country according to alphabetical order. ^a^ 183 *Enterobacter* spp. isolates were not tested. ^b^ Of the 122 isolates, 109 were *K. pneumoniae*, and 13 were *E. coli.* ^c^ All isolates co-carried the bla KPC and blaOXA-48 genes. ^d^ Austria, Belgium, Bulgaria, Croatia, Cyprus, the Czech Republic, Denmark, Estonia, France, Germany, Greece, Hungary, Ireland, Israel, Italy, Latvia, Lithuania, Luxembourg, Malta, Montenegro, North Macedonia, Norway, Poland, Portugal, Romania, Serbia, Slovakia, Slovenia, Spain, Turkey, and the UK. ^e^ Rectal swabs were collected as part of colonization screening. ^f^ Data available for KPC-positive isolates. ^g^ Not specifically reporting countries; SENTRY and MYSTIC surveillance programs. ^h^ Seven isolates collected from urine, three from sputum, and three from miscellaneous sources were considered as colonization. ^i^ Only 371 isolates were tested for the specific *bla*_KPC_-producing genes. ^j^ *Proteus mirabilis* (*n* = 29), *Providencia stuartii* (*n* = 25), and *Morganella morganii* (*n* = 2). ^k^ For 7 isolates, the source of isolation is not reported (NR). ^l^ Argentina, Brazil, Cameroon, Chile, Colombia, Costa Rica, Dominican Republic, Guatemala, Hong Kong, India, Ivory Coast, Jordan, Kuwait, Malaysia, Mexico, Morocco, Nigeria, Panama, the Philippines, Qatar, Russia, Saudi Arabia, South Africa, Taiwan, Thailand, Turkey, and Venezuela. ^m^ 7446 isolates were eligible for beta-lactamase gene screening. ^n^ Not specifically reporting countries; ATLAS surveillance program. ^o^ Amplicon sequencing was not performed in the *bla*_KPC-like_ isolates. These isolates were tested using a multiplex reaction and reamplified using singleplex. ^p^ Austria, Belgium, the Czech Republic, Denmark, France, Germany, Greece, Hungary, Italy, Netherlands, Poland, Portugal, Romania, Russia, Spain, Sweden, Turkey, the United Kingdom, Australia, China, Hong Kong, Japan, Malaysia, the Philippines, South Korea, Taiwan, Thailand, Israel, Kenya, Kuwait, Nigeria, South Africa, and the United States of America.

**Table 2:** Antimicrobial resistance percentages (%) of Klebsiella pneumoniae carbapenemase (KPC)-producing Gram-negative isolates in the included studies.

Study, Year/% [ref.]	CAZ	P/T	ATM	GEN	IMP	MER	COL	TGC
Campos, 2017 ^a^ [111]	90.7	na	na	62	78.7	92.2	na	na
Castanheira, 2012 ^b^ [124]	na	94|100 ^c^	na	45|51 ^c^	na	84|53	na	na
Endimiani, 2009 [98]	100	na	na	na	100	100	na	na
Fang, 2019 [20]	100	100	100	na	100	na	na	na
Ge, 2024 [21]	100	100	na	na	na	100	na	na
Gracia-Ahufinger, 2023 [91]	96	95	72	74	77	85	14	na
Han, 2020 [22]	98	99	99	84	99	98	na	0.4
Hawser, 2009 [131]	na	na	na	na	80.8	80.8	na	0
Kaiser, 2013 [101]	99	100	100	41	100	99	16	1
Karlowsky, 2022 [132]	97|100 ^c^	na	na	na	94|91 ^c^	97|78 ^c^	na	na
Kazmierczak, 2019 [134]	na	na	na	na	na	100	40	na
Logan, 2019 [102]	na	18 [2/11]	0 [0/8]	72	11 ^d^	11	na	na
Nobrega, 2023 [127]	92	97	na	na	na	96	na	na
Santino, 2013 [75]	100	na	na	80	100	100	80	73
Shortridge, 2023 [104]	92.2	96.1	na	35	98.9	86.1	na	2.2
Sorovou, 2023 ^e^ [70]	na	na	18	6	na	na	2	10
Sorovou, 2023 ^f^ [70]	na	na	17	2	na	na	0	10
Sorovou, 2023 ^g^ [70]	na	na	17	11	na	na	7	11
Sorovou, 2023 ^h^ [70]	na	na	59	49	na	na	3	43
Tavares, 2015 [114]	87	na	98	42	na	na	na	47 ^i^
Tian, 2018 [35]	100	na	na	80	100	100	0	0
Tolentino, 2019 [115]	100	100	na	91.7	97.9	100	na	na
Wang, 2020 [39]	na	na	na	na	na	na	na	na
Wang, 2022 [38]	na	na	na	na	100	100	na	na
Wise, 2023 [136]	na	na	99	50	98	96	na	5

Abbreviations: ATM, aztreonam; CAZ, ceftazidime; COL, colistin; GEN, gentamycin; IMP, imipenem; MER, meropenem; P/T, piperacillin–tazobactam; TGC, tigecycline. ^a^ Percentages are a result of the average resistance percentage taken from 3 different hospitals, namely BACG—Beneficent Association of Campo Grande, RHMS—Regional Hospital of Mato Grosso do Sul, and UH/FUMS—Maria Aparecida Pedrossian University Hospital of Federal University of Mato Grosso do Sul. ^b^ Resistance of carbapenemase-producing *Enterobacteriaceae.* ^c^ The first value refers to the percentage of antimicrobial resistance as defined by the CLSI criteria; the second value refers to the percentage of antimicrobial resistance as defined by the EUCAST criteria. ^d^ Antimicrobial resistance reported at 11% as a sum for carbapenems (either imipenem or meropenem). ^e^ Isolates collected in 2019. ^f^ Isolates collected in 2020. ^g^ Isolates collected in 2021. ^h^ Isolates collected in 2022. ^i^ MIC_50_ = 1 mg/L.

## Data Availability

The original contributions presented in the study are included in the article/Supplementary Material, further inquiries can be directed to the corresponding author.

## Supplementary Materials

The following supporting information can be downloaded at: https://www.mdpi.com/article/10.3390/microorganisms13071697/s1, Table S1: Search strings used for each resource to identify relevant articles, done on 20 February 2025. Table S2: Antimicrobial resistance percentages (%) of Klebsiella pneumoniae carbapenemase (KPC)-producing Gram-negative isolates in the included studies.

## Abbreviations

CLSI	Clinical and Laboratory Standards Institute
EUCAST	European Committee on Antimicrobial Susceptibility Testing
HAP	healthcare-associated pneumonia
KPC	Klebsiella pneumoniae carbapenemase
MBL	metallo-β-lactamase
NDM	New Delhi metallo-β-lactamase
PCR	polymerase chain reaction
SSTI	skin and soft tissue infection
ST	sequence type
UTI	urinary tract infection
VAP	ventilator-associated pneumonia
