# Supplementary material for: Global Epidemiology and Antimicrobial Resistance of Klebsiella Pneumoniae Carbapenemase (KPC)-Producing Gram-Negative Clinical Isolates: A Review
Source: Microorganisms. 2025 Jul 19;13(7):1697. doi: 10.3390/microorganisms13071697 (PMC12300886; doi:10.3390/microorganisms13071697)
Supplement: Supplementary file 1 [file microorganisms-13-01697-s001.zip › microorganisms-3703270-Supplementary table S1.pdf]

Supplementary Table S1. Search strings used for each resource to identify relevant articles, done on 20 February 2025.

Resource	Search strategy	Coverage (years)	Results
Embase	(kpc OR "klebsiella pneumoniae carbapenemase") AND (bacteria OR isolates OR pathogens) AND (worldwide OR international OR global) AND (epidemiology OR prevalence OR dissemination OR surveillance)	1974 – 2025	1,436
Web of Science	(kpc OR "klebsiella pneumoniae carbapenemase") AND (bacteria OR isolates OR pathogens) AND (worldwide OR international OR global) AND (epidemiology OR prevalence OR dissemination OR surveillance)	1900 – 2025	1,192
PubMed	(kpc OR "klebsiella pneumoniae carbapenemase") AND (bacteria OR isolates OR pathogens) AND (worldwide OR international OR global) AND (epidemiology OR prevalence OR dissemination OR surveillance)	1946 – 2025	919
Scopus	(kpc OR "klebsiella pneumoniae carbapenemase") AND (bacteria OR isolates OR pathogens) AND (worldwide OR international OR global) AND (epidemiology OR prevalence OR dissemination OR surveillance)	1788 – 2025	584
Google Scholar	(kpc OR "klebsiella pneumoniae carbapenemase") AND (bacteria OR isolates OR pathogens) AND (worldwide OR international OR global) AND (epidemiology OR prevalence OR dissemination OR surveillance)	NA	5,120[a]
Cochrane Library	(kpc OR "klebsiella pneumoniae carbapenemase") AND (bacteria OR isolates OR pathogens) AND (worldwide OR international OR global) AND (epidemiology OR prevalence OR dissemination OR surveillance)	1993 – 2025	10

[a] for Google Scholar only the first 1,000 articles of the 5,120 results could be accessed
