# Supplementary material for: Global Epidemiology and Antimicrobial Resistance of Klebsiella Pneumoniae Carbapenemase (KPC)-Producing Gram-Negative Clinical Isolates: A Review
Source: Microorganisms. 2025 Jul 19;13(7):1697. doi: 10.3390/microorganisms13071697 (PMC12300886; doi:10.3390/microorganisms13071697)
Supplement: Supplementary file 1 [file microorganisms-13-01697-s001.zip › microorganisms-3703270-Supplementary Table S2.pdf]

Supplementary Table S2. Antimicrobial resistance percentages (%) of *Klebsiella pneumoniae* carbapenemase (KPC)-producing Gram-negative isolates in the included studies.

<table>
<tr><th>Study, year / %</th><th>CTX</th><th>CRO</th><th>CAZ</th><th>FEP</th><th>SXT</th><th>P/T</th><th>CFS</th><th>CZA</th><th>ATM</th><th>GEN</th><th>IMP</th><th>MER</th><th>ETP</th><th>COL</th><th>PMB</th><th>TGC</th><th>CIP</th><th>LEV</th></tr>
<tr><td>Campos, 2017[a]</td><td>na</td><td>95.7</td><td>90.7</td><td>85</td><td>na</td><td>na</td><td>na</td><td>na</td><td>na</td><td>62</td><td>78.7</td><td>92.2</td><td>90</td><td>na</td><td>na</td><td>na</td><td>na</td><td>na</td></tr>
<tr><td>Castanheira, 2012[b]</td><td>na</td><td>94</td><td>na</td><td>na</td><td>na</td><td>94|100[c]</td><td>na</td><td>na</td><td>na</td><td>45|51[c]</td><td>na</td><td>84|53[c]</td><td>na</td><td>na</td><td>na</td><td>na</td><td>na</td><td>59|63[c]</td></tr>
<tr><td>Endimiani, 2009</td><td>na</td><td>100</td><td>100</td><td>na</td><td>100</td><td>na</td><td>na</td><td>na</td><td>na</td><td>na</td><td>100</td><td>100</td><td>100</td><td>na</td><td>na</td><td>na</td><td>100</td><td>na</td></tr>
<tr><td>Fang, 2019</td><td>na</td><td>na</td><td>100</td><td>na</td><td>na</td><td>100</td><td>100</td><td>na</td><td>100</td><td>na</td><td>100</td><td>na</td><td>na</td><td>na</td><td>na</td><td>na</td><td>na</td><td>100</td></tr>
<tr><td>Ge, 2024</td><td>na</td><td>na</td><td>100</td><td>na</td><td>na</td><td>100</td><td>100</td><td>na</td><td>na</td><td>na</td><td>na</td><td>100</td><td>na</td><td>na</td><td>na</td><td>na</td><td>na</td><td>na</td></tr>
<tr><td>Gracia-Ahufinger, 2023</td><td>100</td><td>na</td><td>96</td><td>93</td><td>na</td><td>95</td><td>na</td><td>na</td><td>72</td><td>74</td><td>77</td><td>85</td><td>99</td><td>14</td><td>na</td><td>na</td><td>92</td><td>na</td></tr>
<tr><td>Han, 2020</td><td>na</td><td>99</td><td>98</td><td>98</td><td>48</td><td>99</td><td>98</td><td>na</td><td>99</td><td>84</td><td>99</td><td>98</td><td>99</td><td>na</td><td>4</td><td>0.4</td><td>96</td><td>95</td></tr>
<tr><td>Hawser, 2009</td><td>na</td><td>na</td><td>na</td><td>na</td><td>na</td><td>na</td><td>na</td><td>na</td><td>na</td><td>na</td><td>80.8</td><td>80.8</td><td>96.2</td><td>na</td><td>na</td><td>0</td><td>na</td><td>na</td></tr>
<tr><td>Kaiser, 2013</td><td>na</td><td>100</td><td>99</td><td>72</td><td>90</td><td>100</td><td>na</td><td>na</td><td>100</td><td>41</td><td>100</td><td>99</td><td>100</td><td>16</td><td>na</td><td>1</td><td>90</td><td>na</td></tr>
<tr><td>Karlowski, 2022</td><td>na</td><td>na</td><td>97|100[c]</td><td>91|94[c]</td><td>83|80[c]</td><td>na</td><td>na</td><td>na</td><td>na</td><td>na</td><td>94|91[c]</td><td>97|78[c]</td><td>na</td><td>na</td><td>na</td><td>na</td><td>na</td><td>84|84[c]</td></tr>
<tr><td>Kazmierczak, 2019</td><td>na</td><td>na</td><td>na</td><td>93</td><td>na</td><td>na</td><td>na</td><td>na</td><td>na</td><td>na</td><td>na</td><td>100</td><td>na</td><td>40</td><td>na</td><td>na</td><td>91</td><td>na</td></tr>
<tr><td>Logan, 2019</td><td>na</td><td>na</td><td>na</td><td>na</td><td>5.6</td><td>18
[2/11]</td><td>na</td><td>na</td><td>0
[0/8]</td><td>72</td><td>11[d]</td><td>11</td><td>na</td><td>na</td><td>na</td><td>na</td><td>23[e]
[3/13]</td><td>23
[3/13]</td></tr>
<tr><td>Nobrega, 2023</td><td>na</td><td>93</td><td>92</td><td>na</td><td>59</td><td>97</td><td>na</td><td>na</td><td>na</td><td>na</td><td>na</td><td>96</td><td>na</td><td>na</td><td>na</td><td>na</td><td>na</td><td>na</td></tr>
<tr><td>Santino, 2013</td><td>na</td><td>na</td><td>100</td><td>na</td><td>100</td><td>na</td><td>na</td><td>na</td><td>na</td><td>80</td><td>100</td><td>100</td><td>na</td><td>80</td><td>na</td><td>73</td><td>100</td><td>100</td></tr>
<tr><td>Shortridge, 2023</td><td>na</td><td>na</td><td>92.2</td><td>78.3</td><td>na</td><td>96.1</td><td>na</td><td>1.6[f]</td><td>na</td><td>35</td><td>98.9</td><td>86.1</td><td>na</td><td>na</td><td>na</td><td>2.2</td><td>na</td><td>79.4</td></tr>
<tr><td>Sorovou, 2023[g]</td><td>na</td><td>na</td><td>na</td><td>na</td><td>10</td><td>na</td><td>na</td><td>4</td><td>18</td><td>6</td><td>na</td><td>na</td><td>na</td><td>2</td><td>na</td><td>10</td><td>na</td><td>na</td></tr>
<tr><td>Sorovou, 2023[h]</td><td>na</td><td>na</td><td>na</td><td>na</td><td>17</td><td>na</td><td>na</td><td>2</td><td>17</td><td>2</td><td>na</td><td>na</td><td>na</td><td>0</td><td>na</td><td>10</td><td>na</td><td>na</td></tr>
<tr><td>Sorovou, 2023[i]</td><td>na</td><td>na</td><td>na</td><td>na</td><td>15</td><td>na</td><td>na</td><td>0</td><td>17</td><td>11</td><td>na</td><td>na</td><td>na</td><td>7</td><td>na</td><td>11</td><td>na</td><td>na</td></tr>
</table>

Sorovou, 2023[j]	na	na	na	na	52	na	na	0	59	49	na	na	na	3	na	43	na	na
Tavares, 2015	100[k]	na	87	83	61	na	na	na	98	42	na	na	na	na	17[l]	47[m]	86	na
Tian, 2018	100	na	100	na	87	na	100	na	na	80	100	100	100	0	na	0	97	97
Tolentino, 2019	na	100	100	100	na	100	na	na	na	91.7	97.9	100	100	na	na	na	95.8	na
Wang, 2020	na	na	na	na	na	na	100	na	na	na	na	na	100	na	na	na	na	na
Wang, 2022	na	100	na	na	na	na	100	na	na	na	100	100	na	na	na	na	na	na
Wise, 2023	na	na	na	96	na	na	na	1	99	50	98	96	na	na	na	5	na	86

Abbreviations: ATM, aztreonam; CAZ, ceftazidime; CFS, cefoperazone-sulbactam; CIP, ciprofloxacin; CRO, ceftriaxone; CTX, cefotaxime; CZA, ceftazidime-avibactam; COL, colistin; ETP, ertapenem; FEP, cefepime; GEN, gentamycin; IMP, imipenem; LEV, levofloxacin; MER, meropenem; PMB, polymyxin B; P/T, piperacillin-tazobactam; SXT, cotrimoxazole; TGC, tigecycline

[a] Percentages are a result of the average resistance percentage taken from 3 different hospitals: BACG-Beneficent Association of Campo Grande, RHMS-Regional Hospital of Mato Grosso do Sul, UH/FUMS-Maria Aparecida Pedrossian University Hospital of Federal University of Mato Grosso do Sul

[b] Resistance of carbapenemase-producing *Enterobacteriaceae*

[c] The first value refers to the percentage of antimicrobial resistance as defined by the CLSI criteria; the second value refers to the percentage of antimicrobial resistance as defined by the EUCAST criteria

d Antimicrobial resistance reported at 11% as a sum for carbapenems (either imipenem or meropenem)

[e] Antimicrobial resistance reported at 11% as a sum for fluoroquinolones (ciprofloxacin and levofloxacin)

f In total, 126 isolates were tested for ceftazidime-avibactam (CZA)

g Isolates collected in 2019

[h] Isolates collected in 2020

[i] Isolates collected in 2021

[j] Isolates collected in 2022

[k] Percentage of resistant or intermediate resistant strains using the disk diffusion method

[l] After excluding intrinsically resistant strains, MIC_{50} = 0.75 mg/L

[m] MIC_{50} = 1 mg/L
